# Supplementary material for: A Critical View of the Application of the APEX Software (Aqueous Photochemistry of Environmentally-Occurring Xenobiotics) to Predict Photoreaction Kinetics in Surface Freshwaters
Source: Molecules. 2019 Dec 18;25(1):9. doi: 10.3390/molecules25010009 (PMC7017383; doi:10.3390/molecules25010009)
Supplement: Supplementary file 1 [file molecules-25-00009-s001.zip › 3.2.4_gcc-4.4.0.zip!/3.2.4_gcc-4.4.0/3.2.4_gcc-4.4.0/doc/octave/html/interpreter/Data-Structures.html]

Data Structures - Untitled


Next: Cell Arrays,
Up: Data Containers


---

### 6.1 Data Structures

Octave includes support for organizing data in structures. The current
implementation uses an associative array with indices limited to
strings, but the syntax is more like C-style structures.

- Basic Usage and Examples- Structure Arrays- Creating Structures- Manipulating Structures- Processing Data in Structures
